# Editorial: Women in regulatory toxicology: 2021

**DOI:** 10.3389/ftox.2022.1056285

**Published:** 2022-10-18

**Authors:** Marlene Ågerstrand, Anna Beronius, Marion Junghans, Olwenn Martin

**Affiliations:** ^1^ Department of Environmental Science, Stockholm University, Stockholm, Sweden; ^2^ Institute of Environmental Medicine, Karolinska Institutet, Stockholm, Sweden; ^3^ Swiss Centre for Applied Ecotoxicology Eawag-EPFL, Dübendorf, Switzerland; ^4^ Department of Arts and Science, University College London, London, United Kingdom

**Keywords:** gender bias, women in science, regulatory toxicology, regulatory science, women in STEM

Less than 30% of researchers worldwide are women (UNESCO, 2019) and even if some countries display more promising numbers it remains difficult for women to obtain senior positions, a phenomenon called the “leaky pipeline” (e.g., Office fédéral de la statistique 2021). Long-standing biases and gender stereotypes are discouraging women away from science-related fields, and Science, Technology, Engineering and Math (STEM) research in particular. Key aspects to reduce the gender gap include combating stereotypes and biases of what a researcher is and may look like, moving away from a work environment where discrimination, sexual harassment, and other illegal behaviours are present, and cultivating a sense of belonging among women (O’Connell and McKinnon, 2021). The available literature suggests that female role models can play an important role in women’s STEM motivation (Herrmann et al., 2016; O’Brien et al., 2017; Gladstone and Cimpian, 2021; Zhang and Rios, 2022). That, in combination with that gender equality is essential to ensure sustainable development (UN Women, 2022), inspired us to support this Research Topic on Women in Regulatory Toxicology. While the bulk of chemical production was historically emanating from Europe and the US, and therefore most regulatory models conceptualised in Western economies, this has now shifted to emerging economies such as China, India and Brazil (UNEP, 2019). In this context, it is not only important to include the voices of women in regulatory toxicology, but equally crucial that women from the Global South be represented.

“Regulatory science consists of an applied version of various scientific disciplines used in the regulatory process” (Moghissi et al., 2014). Regulatory science in the field of toxicology is the application of quantitatively and/or qualitatively scientific methods for the development of new methods, tools, and approaches for the assessment and management of chemicals, and it is the analysis and evaluation of the outcome of regulatory processes and policy developments. Thereby, Regulatory science has the potential to strengthen the protection of human health and the environment from hazardous chemicals. We appreciate the increasing gender awareness in regulatory sciences and risk assessment (e.g., Holthaus, 2019) and would like to encourage all regulatory scientists to contribute to ensuring risk assessment guidance is protective of all genders (e.g., Later et al., 2010). The contributions to this Research Topic do not address gender issues in Regulatory Science, instead, it is a celebration of women’s contribution to STEM: high-quality research of significant importance to society. The six contributions, written by 23 authors of which 19 are women, include three original research articles and three perspectives which all explore different aspects of regulatory toxicology, highlighting the difficulties and possibilities with the use of new methodologies in hazard and risk assessment, as well as evaluations of current and past regulatory initiatives.

The first research article makes an important point about regulatory foresight in considering how chemical safety can guide material innovations at the design stage (Harper et al.). *In vitro* estrogen, androgen and progestagen reporter gene assays were coupled with migration testing to inform the development of safer and sustainable packaging bio-based alternatives to petroleum-based plastics. The second research article presents an original take on New Approach Methodologies (NAMs) (Ponder et al.). The authors capitalised on published clinical evidence by developing an *in litero* method to establish a reference list of respiratory sensitisers, thereby demonstrating the benefits of thinking beyond disciplinary silos to meet unmet regulatory needs. In the last research article, the identification of substances of concern in food is performed by combining exposure data with the Threshold of Toxicological Concern (TTC), where new approach methodologies CNAMs) are encouraged, followed by high-throughput screening (HTS) approaches (Luijten et al.). This is suggested as a pragmatic approach to prioritising those substances with the highest risk for the actual risk assessments.

The first perspective article proposes an Adverse Outcome Pathway (AOP) describing how interaction by nano-sized particles with components in the lung resident cell membrane may be causally linked to the development of lung cancer (Nymark et al.). The authors also discuss the potential for using the AOP as a basis for the development of integrated *in silico*- and *in vitro*-based testing strategies using standardized New Approach Methodologies (NAMs). This work is of high regulatory relevance given the rise of nanotechnology and accompanying concern of potential health risks, as well as demonstrating the potential application of AOP methodology and NAMs in the regulatory setting. The second perspective discusses challenges in the regulatory acceptance of NAMs in Brazilian legislation for the registration of pharmaceuticals, medical devices, food/supplements, and agrochemical products (Villela and Machado). While the use of data from NAMs is generally promoted, regulatory acceptance and uptake are slow. The authors discuss factors that hamper this process, including uncertainties regarding data interpretation and their relevance for risk assessment, as well as perceived limitations in methodology and reporting. The article highlights the importance of collaboration and joint efforts between regulators, industry, CROs, and researchers to build regulatory confidence in the use of NAMs. In the last perspective, the implementation of chemical regulation, which has proven to be challenging for various reasons, is examined (Maffini and Vandenberg). In the U.S., 25 years have passed since congress passed a law stating that pesticides used in food should be tested for endocrine disruption. Still, only a handful of substances have been tested, none have been identified as endocrine disruptors, and there have been no regulatory actions to reduce or prevent exposure. After a thorough analysis of the available documents, the authors conclude that the U.S. EPA has failed in its implementation of the law, thereby putting human health and the environment at risk.

To change traditional mindsets, gender equality in science must be encouraged. Promoting our fellow women colleagues, sharing our networks, and being an ally are examples of what can be done to overcome these barriers.

We wish to offer our gratitude to the authors and reviewers that contributed to this Research Topic.
